# Design and Characterization of Chitosan-Based Smart Injectable Hydrogel for Improved Sustained Release of Antinarcotics

**DOI:** 10.3390/ph17060749

**Published:** 2024-06-07

**Authors:** Maryam Aftab, Fatima Javed, Sajjad Haider, Rawaiz Khan, Salah Uddin Khan, Kamran Alam, Afreenish Amir, Faheem Ullah, Naseer Ali Shah

**Affiliations:** 1 Department of Biosciences, COMSATS University, Park Road, Islamabad 45520, Pakistan; 2 Department of Chemistry, Shaheed Benazir Bhutto Women University, Peshawar 25000, Pakistan; fatimajaved@ymail.com; 3 Department of Chemical Engineering, King Saud University, Riyadh 11545, Saudi Arabia; shaider@ksu.edu.sa; 4 Restorative Dental Sciences Department, College of Dentistry, King Saud University, Riyadh 11545, Saudi Arabia; kraez@ksu.edu.sa; 5 College of Engineering, King Saud University, P.O. Box 800, Riyadh 11421, Saudi Arabia; drskhan@ksu.edu.sa; 6 Separation and Conversation Technology, Flemish Institute for Technological Research (VITO), 2400 Mol, Belgium; kamran.alam@vito.be; 7 Department of Microbiology, National Institute of Health, Islamabad 45500, Pakistan; afreenish.amir@nih.org.pk; 8 Department of Biological Sciences, National University of Medical Sciences (NUMS), Rawalpindi 46000, Pakistan; 9 School of Materials and Mineral Resources Engineering, Engineering Campus, University Sains Malaysia, Nibong Tebal 14300, Malaysia

**Keywords:** injectable hydrogel, chitosan, sustained release, naltrexone, disulfiram, ethanol

## Abstract

The treatment adherence of narcotics-addicted individuals with reduced incidences of relapse can be enhanced by a sustained drug release formulation of antinarcotics. So far, different drug formulations have been reported with sustained drug release periods of 28 and 35 days. To further enhance this duration, different formulations of injectable hydrogels (IHs) have been developed by combining low molecular weight (LMW) and high molecular weight (HMW) chitosan (CS) with guar gum (GG) and crosslinking them by sodium bi phosphate dibasic. The structural, morphological, and physicochemical properties of LMW-CS IH, and HMW-CS IH were evaluated using Fourier transform infrared spectroscopy (FT-IR), thermo-gravimetric analysis (TGA), scanning electron microscopy (SEM), and rheological, swelling, and biodegradation analysis. The HMW-CS IH showed high crosslinking, increased thermal stability, high mechanical strength, elevated swelling, and low biodegradation. The antinarcotic drugs naltrexone (NTX) and disulfiram (DSF) were loaded separately into the HMW-CS IH and LMW-CS IH. The release of NTX and DSF was investigated in phosphate buffer saline (PBS) and ethanol (0.3%, 0.4%, and 0.5%) over a 56-day period using an UV spectrophotometer. The drug release data were tested in zero-order, first-order, and Korsemeyer–Peppas mathematical models. In PBS, all prepared formulations followed non-Fickian drug release, while in ethanol, only NTX HMW-CS IH followed non-Fickian release in all three different concentrations of ethanol.

## 1. Introduction

Researchers are exploring novel solutions to remedy addiction due to the euphoric and addictive potential of narcotics, including opioids and alcohol. The addictive and dependency-inducing nature of ethanol, a psychoactive compound found in alcoholic beverages, has been widely recognized [1]. The relationship between ethanol use and addiction involves complex interactions among environmental, behavioral, and biological factors [2]. Symptoms of alcohol intoxication are signaled by a blood alcohol concentration (BAC) of 0.3%, severe alcohol intoxication by 0.4% BAC, and a potentially fatal BAC of 0.5% can lead to coma [3].

Disulfiram (DSF) and naltrexone (NTX) are FDA-approved medicines for alcohol use disorder. They are found to be equally efficient in decreasing cravings and the prevention of addiction relapse [4]. DSF is employed for the treatment of alcohol use disorders as it inhibits the enzyme aldehyde dehydrogenase, which is responsible for metabolizing alcohol by converting acetaldehyde (a byproduct of alcohol) into acetate. DSF inhibits acetaldehyde metabolism, leading to acetaldehyde buildup. To achieve therapeutic effects, DSF needs to be administered regularly [5]. NTX is a non-selective opioid antagonist with a strong affinity for mu-opioid receptors. Its effects are extended by its metabolite 6-beta naltrexol [6]. NTX decreases the desire for addictive substances by lowering dopamine levels, which control the pleasurable effects of alcohol and other abusive substances, whether administered orally or by injection [7,8]. 

Although NTX and DSF have been found to be highly effective for the treatment of narcotics addiction, adherence to treatment is still a major challenge. Oral antinarcotics reduce treatment retention and increase addiction relapse [9]. Over the past decade, extended-release formulations have become essential for addiction therapy [10]. The available sustained release dosage forms can release NTX up to 28 days from micro emulsions [11] and 35 days from fluid crystal injections [12]. The sustained release effect of DSF with available dosage forms has been observed to be 24 h from nano emulsion gels in situ [13] and 14 days from thermosensitive hydrogels [14]. The chronic nature of narcotics addiction affords frequent relapse and long-term treatment is obligatory to achieve treatment goals. The development of new formulations can contribute towards long-term treatment success. Patients require extended-release treatment with single doses of pharmaceutical dosage forms [15,16]. 

Hydrogels comprise a three-dimensional (3D) network that can absorb a large amount of water and undergoes swelling due to hydrophilic groups, such as amino, hydroxyl, carboxyl, and amide groups. The network of a hydrogel is constructed by the crosslinking of polymer chains. Hydrogels, being flexible and soft, can be designed by chemical or physical crosslinking of natural or synthetic polymers [17]. The mechanical strength exhibited in crosslinked hydrogel is important for pharmaceutical and biomedical applications [18]. The mechanical strength of hydrogel is important for the design of drug delivery matrixes as it sustains the physical structure during the release of the therapeutic entity for a specified time period [19]. The injectable hydrogels provide unique advantages due to their property of injectability. Injectable hydrogels can be administered to various sites in the body with reduced discomfort at the site of administration. They impart a versatile platform in the fields of drug delivery and tissue engineering applications [20]. 

Some formulations of injectable hydrogel (IH) can address the extended and sustained release of NTX and DSF with minimal dose frequency [21]. Biodegradable and biocompatible polymers are commonly used to prepare IH for the safe delivery of medicinal substances within the body [22]. Chitosan (CS) is a naturally derived polymer known for its superior biodegradability, biocompatibility, biocidal properties, and minimal immunogenic response [23]. The molecular weight (MW) of CS significantly influences drug encapsulation, loading, release characteristics, and sustained drug delivery. An increase in MW leads to the formation of more inter-chain networks, resulting in improved thermal, mechanical, physicochemical, and stability properties [24]. Furthermore, guar gum (GG), a natural polymer with various properties such as emulsification, thickening, drug delivery, bulking, and antibacterial and antioxidant effects, is preferred for the preparation of IH [25]. The synergy of CS and GG results in strong electrostatic contacts, improved complex formation, increased swelling, and favorable physicochemical properties, making them ideal choices for drug delivery.

The current study aimed to develop an IH based on the MW of CS crosslinked to GG by sodium phosphate dibasic. Following characterization of HMW-CS IH and LMW-CS-IH, DSF and NTX were separately encapsulated in the interpenetrating IH. The release of NTX and DSF was investigated in PBS and lethal alcohol concentrations (0.3%, 0.4%, and 0.5%) over a period of two months. Drug release kinetic models were applied to evaluate the mechanism of NTX and DSF’s release from IH upon exposure to an in vitro release medium. The NTX and DSF exert their potential to counter narcotics by acting as an opioid receptor antagonist and inhibiting the enzyme aldehyde dehydrogenase, respectively. Both medications have been proven effective in treating alcohol abuse [4,10]. Ethanol concentrations of 0.3%, 0.4%, and 0.5% were used to create an environment for the NTX- and DSF-loaded HMW-CS IH and LMW-CS IH that mimicked lethal blood alcohol concentrations. This allowed for the effective release of antinarcotics to treat high levels of alcohol abuse 

## 2. Results and Discussion

### 2.1. Preparation of Hydrogel

**Figure 1:**
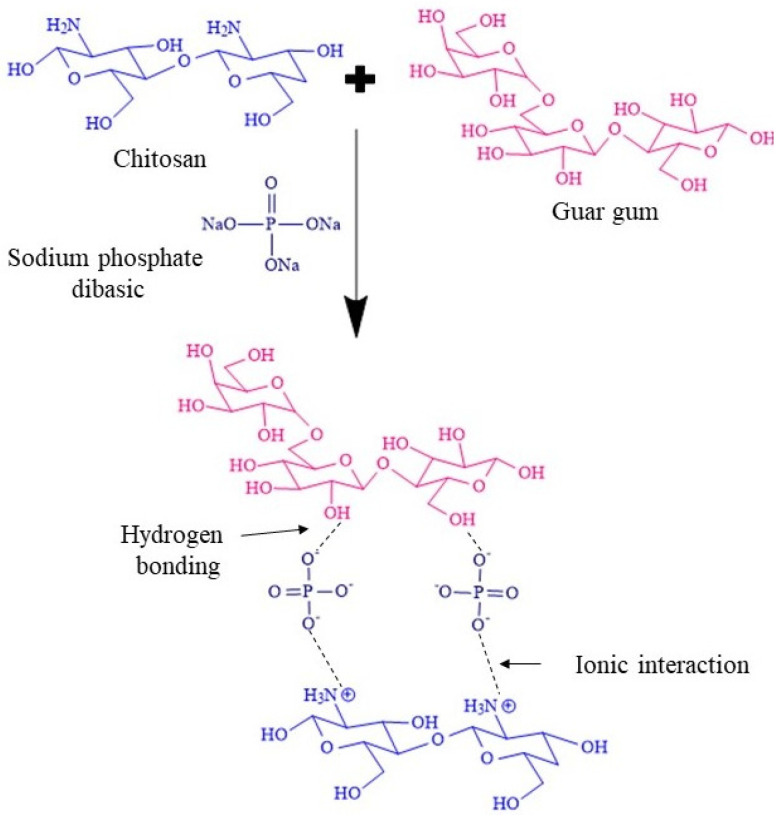
Synthetic scheme for the crosslinking between CS and GG, where sodium phosphate dibasic induces physical crosslinking across the polymeric chain by hydrogen bonds and ionic interactions.

HMW- and LMW-CS differ in molecular interactions in terms of their degree of deacetylation. HMW-CS possesses a high number of active amino groups, while in LMW-CS, deacetylation is lower and the amount of active amino groups is fewer [26]. The amino groups of CS become protonated in acidic conditions and form ionic interactions with the negatively charged phosphate ions of the sodium phosphate dibasic. In addition, the hydroxyl groups of GG form hydrogen bonds with the crosslinker. This process leads to the physical crosslinking between CS and GG, which gave stability to the developed IHs. As a result of these electrostatic interactions, the mechanical strength of the developed IHs was enhanced and a biocompatible system for sustained drug delivery was created. The developed IHs remained stable for a period of three months at room temperature. The crosslinking potential of sodium phosphate dibasic helped maintain the stability of the system without any contamination. The synthetic scheme for the crosslinking between CS and GG is illustrated in Figure 1.

**Figure 2:**
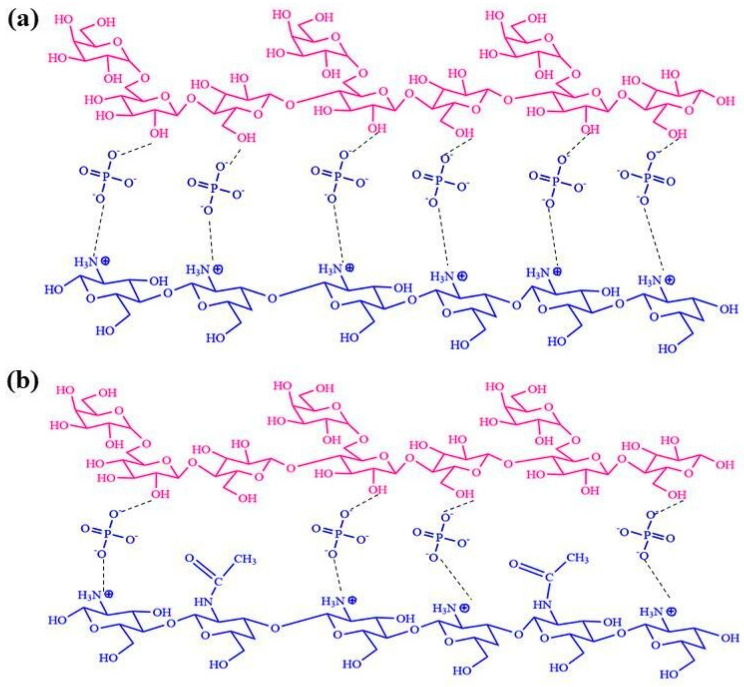
The crosslinking between CS and GG increased with the number of protonated amino groups. (**a**) HMW-CS forms a highly crosslinked structure with GG, while (**b**) LMW-CS, due to the presence of higher acetylation, forms decreased crosslinking with GG.

LMW-CS and HMW-CS were separately crosslinked to GG, and the prepared hydrogels were named HMW-CS IH and LMW-CS IH. The degree of deacetylation was higher in HMW-CS and the crosslinking was increased. The degree of deacetylation was lower in LMW-CS and the crosslinking was lower, as illustrated in Figure 2.

### 2.2. Fourier Transform Infrared (FT-IR) Spectroscopy

**Figure 3:**
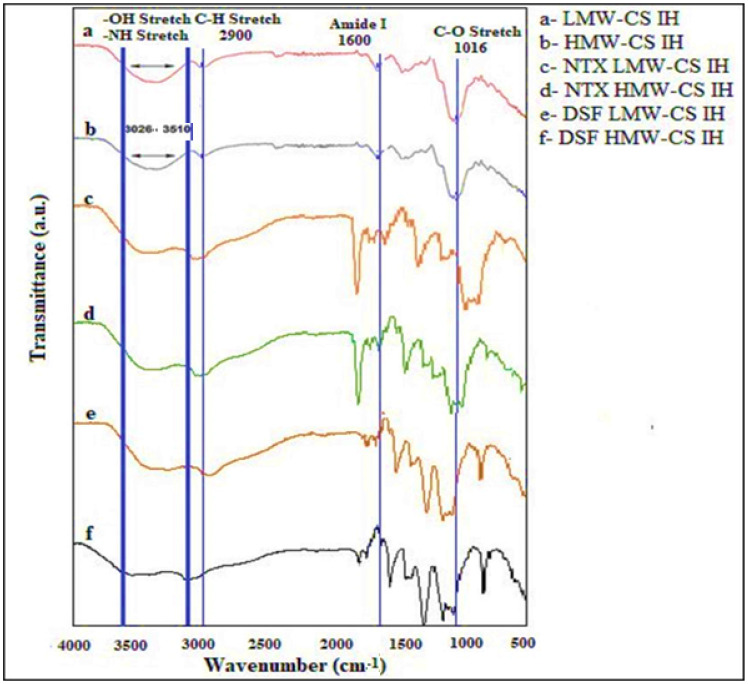
FT-IR spectra: (**a**), LMW-CS IH, (**b**) HMW-CS IH, (**c**) NTX LMW-CS IH, (**d**) NTX-HMW-CS IH, (**e**) DSF-LMW-CS IH, and (**f**) DSF-HMW-CS IH.

The FTIR spectrums of LMW-CS IH and HMW-CS IH with and without encapsulated drugs (NTX and DSF) are illustrated in Figure 3. The FTIR spectra displayed a C-H stretching peak at approximately 2900 cm^−1^. The -OH and -NH stretching bands were seen at approximately 3000–3510 cm^−1^ [27], which showed inter- and intramolecular hydrogen bonding. The peaks at approximately 1600 cm^−1^ and 1016 cm^−1^ were identified as amide I [28] and C-O stretching [29], respectively. The increased intensity of LMW-CS IH was attributed to a greater number of independent resonating C-O stretching bonds with comparatively low stretching. The FTIR spectrum of drug-loaded hydrogel revealed additional peaks for C-N at 1508. A sharp carbonyl peak at 1700 cm^−1^ and aromatic C=C peak at 1617 cm^−1^ were observed due to NTX. The region of 3000–3500 cm^−1^ was broadened after encapsulation of the drug due to the existence of -OH groups [30]. The FT-IR spectra of DSF-loaded LMW-CS IH and HMW-CS IH showed characteristic peaks at 1351–1400 cm^−1^ due to CH_2_-CH_3_ deformations. The peaks observed at 666 cm^−1^ were assigned to S-S dihedral bending in DSF [31]. Thus, the overall spectra clearly indicated the incorporation of the drugs within the hydrogel matrices via the appearance of these additional peaks.

### 2.3. Thermo-Gravimetric Analysis (TGA) 

**Figure 4:**
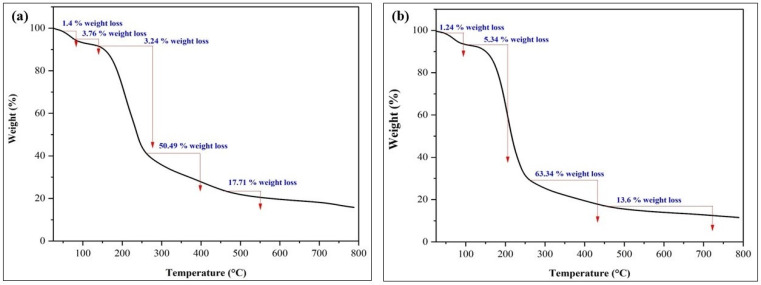
TGA analysis (**a**) HMW-CS IH and (**b**) LMW-CS IH.

All the components of the developed IHs were compatible and their combined thermal effects were utilized to generate the TGA curve of the hydrogel. TGA was used to investigate the mass loss % as a function of temperature for compatibility investigations [32]. The physical crosslinking of the GG and CS in the IHs did not cause any physical degradation and allowed biopolymers to retain their chemical integrity. Thermograms for HMW-CS IH (a) and LMW-CS IH (b) are shown in Figure 4. 

The HMW-CS IH underwent a 50.49% weight loss at a temperature of 400 °C. LMW-CS IH underwent a prominent weight loss of 63.34% at 440 °C. At the end of the analysis, the residual mass was found to be 15.84% for HMW-CS IH and 11.55% for LMW-CS IH. Supare, K. et al. conducted a study on the controlled release of herbicide from starch–CS hydrogels and TGA analysis revealed that hydrogels crosslinked with glutarladehyde were thermally more stable than those crosslinked with glyoxal [33]. In the current study, the thermal stability of the prepared hydrogels was based on the MW of CS. The LMW-CS IH revealed a lower melting point, less thermal stability, and higher permeability than the HMW-CS IH. 

### 2.4. Scanning Electron Microscopy (SEM)

**Figure 5:**
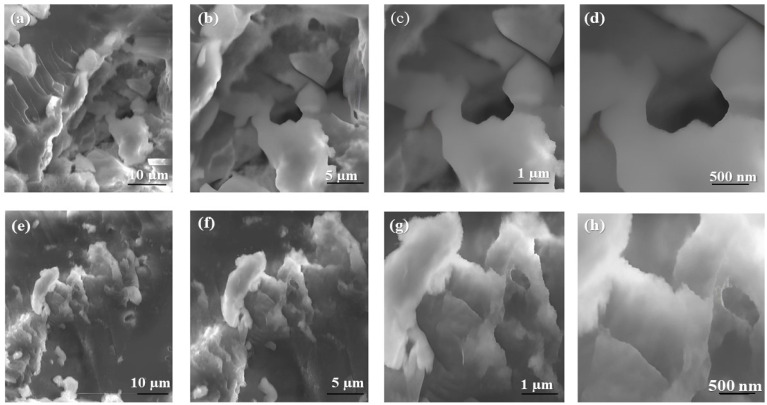
SEM analysis of hydrogels HMW-CS IH (**a**–**d**) and LMW-CS IH (**e**–**h**). The SEM images were taken at resolutions of 10 µm, 5 µm, 1 µm, and 500 nm.

The morphology of the crosslinked and thermally stable IHs were analyzed by SEM. The SEM images were taken at resolutions of 10 µm, 5 µm, 1 µm, and 500 nm. The SEM micrographs, shown in Figure 5, clearly revealed that the prepared hydrogels possessed a porous structure. The SEM images of HMW-CS IH (a, b, c, and d) revealed a denser network structure, enhanced mechanical strength, high swelling capacity, and reduced biodegradation. The open structure created was a result of the electrostatic interactions between the protonated amino groups of CS and hydroxyl groups of GG. The coordinated network enabled the medicines to be incorporated into the hydrogel network and released in a sustained manner [34]. On the contrary, the mechanical strength of the hydrogel decreased with a decrease in the MW of the CS, as observed in the SEM images of LMW-CS IH (e, f, g, and h). The physicochemical parameters, network structure, and framework density improved as the MW of the CS increased [35]. The size of the hydrogel was roughly analyzed to be up to 500 nm, as the SEM images were taken in the dried form. Agglomeration was observed in hydrogel clusters, as clearly depicted in the SEM images. The hydrogel was examined in a highly associated form, and individual particles were not observed.

### 2.5. Rheology

**Figure 6:**
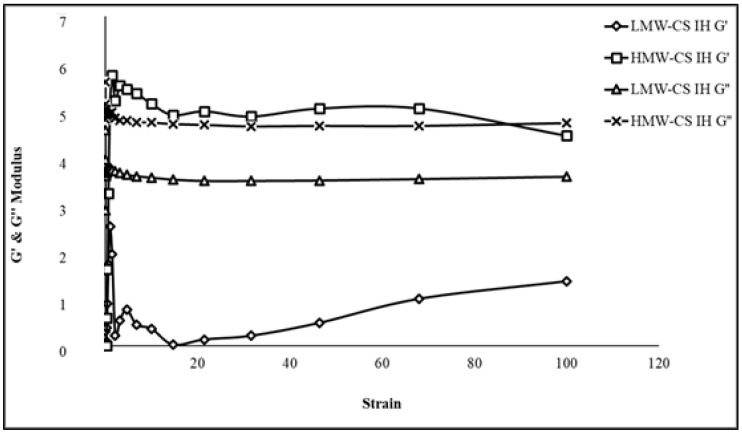
The correlation of storage (G′) and loss modulus (G″) of the HMW-CS IH and LMW-CS IH as a function of angular frequency.

Oscillatory strain–sweep tests were conducted to examine the dynamic rheological characteristics of the IHs. The analysis revealed a linear viscoelastic (LVE) area where the storage modulus (G′) and loss modulus (G″) remained steady regardless of the strain applied, as illustrated in Figure 6. The strain–sweep experiments were performed on fully developed hydrogels, varying from 0.1% to 100% strain amplitude, at a constant frequency of 1.5 Hz and a temperature of 37 °C. The HMW-CS IH and LMW-CS IH exhibited G″ values greater than their G′ values, demonstrating the formation of a viscous gel [36]. 

The LVE zones of both hydrogels extended between 10% to 100% of the applied strain. When the strain exceeded 100%, the hydrogel networks were destroyed. The LMW-CS IH showed liquid-like behavior with G′ < G″ throughout the strain range. The HMW-CS IH revealed ideal rheological characteristics. When the strain reached 100%, the values of G′ and G″ linked and then decreased, suggesting a transition from a gel to a sol state, confirming excellent shear-thinning behavior [37]. Similar rheological analysis was conducted on chitosan hydrogel crosslinked with trimesic acid by Emani, S. et al., 2023. The nature of the gel was analyzed by frequency sweep testing within the limit of the linear viscoelastic region. With the increase in crosslinking degree, the strength of the hydrogel network increased [38].

### 2.6. Swelling Analysis

**Figure 7:**
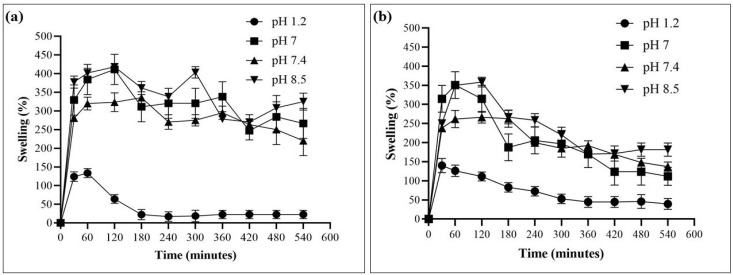
Percentage swelling for (**a**) HMW-CS IH and (**b**) LMW-CS IH at pH 1.2, 7, 7.4, and 8.5.

The swelling of HMW-CS IH and LMW-CS IH was observed at pH 1.2 (gastric pH), 7 (deionized water), 7.4 (physiological pH), and 8.5 (intestinal pH). Equilibrium swelling was attained until 240 min, as depicted in Figure 7. The HMW-CS IH reached a maximum swelling of 400% at pH 8.5 within 120 min, while LMW-CS IH exhibited maximum swelling of 350%. At pH 7, HMW-CS IH exhibited a maximum swelling of 418% within 120 min, while the LMW-CS IH exhibited maximum swelling of 354%. The reduced swelling in the LMW-CS IH was attributed to the diminished coordinated network, weak mechanical strength, and poor inter-chain attraction among pendant groups in the LMW-CS hydrogel [39]. 

Swelling in the prepared IHs was caused by the ionization of amino groups in CS and hydroxyl groups in GG, leading to electrostatic repulsion between the ionized groups at higher pH [40]. With the increase in pH, there was increased formation of hydroxyl groups in the surrounding solution, leading to greater ionization of amino groups in CS and hydroxyl groups in GG. This increased ionization resulted in higher repulsion forces, causing the swelling rate to increase. 

On the contrary, the swelling percentage was significantly lower for HMW-CS IH and LMW-CS IH at pH 1.2. At a lower pH, the ammonium groups of the chitosan backbone are involved in strong ionic interactions with the negatively charged sodium phosphate dibasic and they do not interact with the surrounding protons of the acidic environment. The electrostatic repulsion is lower in acidic environments and swelling is decreased [41]. Rasool, A. et al.; 2019 conducted swelling analysis on CS-based blend hydrogels. The results evaluated that at higher pH, the swelling of CS hydrogels was higher due to increased hydrogen bonding between CS and acrylic acid, while at lower pH the ammonium ions were engaged in positive and negative charge interactions and this prevented the solvent molecules from interacting with the hydrogel and swelling was decreased [42].

### 2.7. Biodegradation Analysis

**Figure 8:**
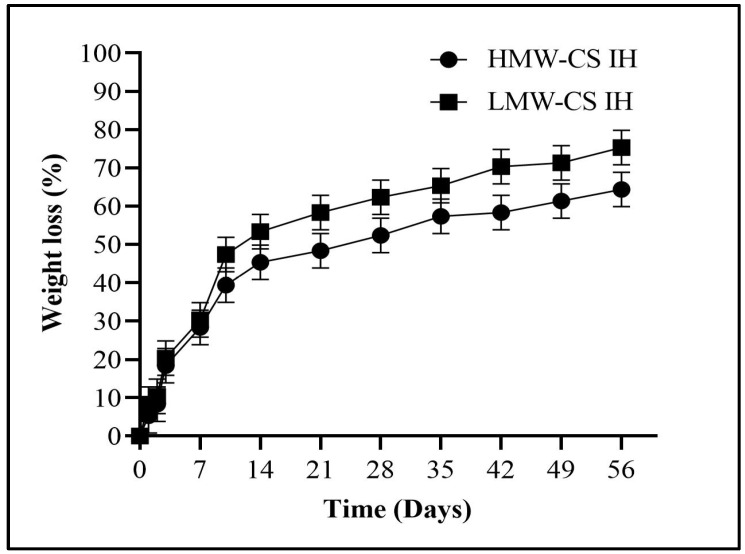
Biodegradation evaluation of HMW- and LMW-CS hydrogels in PBS (pH 7.4 and temperature 37 °C).

The HMW-CS IH and LMW-CS IH were immersed in PBS solution at pH 7.4 and 37 °C. They were placed in a shaking incubator at 100 rpm. Figure 8 clearly illustrates that over a two-month time period, the LMW-CS IH underwent a weight loss of 68%, whereas the HMW-CS IH lost 55% of its initial weight. An increase in the MW of the CS enhanced the mechanical strength and crosslinking network of the IH by promoting more inter- and intramolecular hydrogen bonds. Hence, degradation was less in HMW-CS IH [43]. The less-crystalline structure and lower mechanical strength of LMW-CS IH induced faster degradation [44]. The biodegradation was found to be less in HMW-CS IH due to increased crosslinking [45]. Therefore, the encapsulated drug is released slowly in a sustained manner, which will help to maintain a high rate of treatment adherence for narcotics addiction. Nazir, A., et al.; 2024 explained the mechanism of hydrogel biodegradation based upon the extent of crosslinking. Higher levels of crosslinking increased the network stability of hydrogels and decreased their biodegradation rate, while low crosslinking and high pore size facilitated the degrading agent reaching the polymer chains and inducing degradation earlier [46]. 

### 2.8. Drug Loading

**Figure 9:**
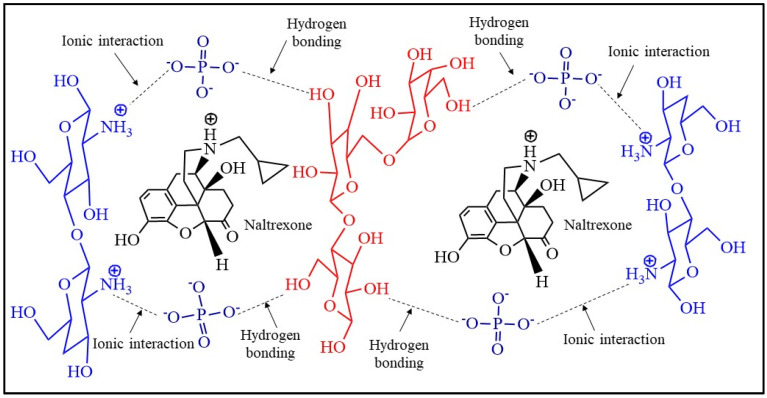
Loading of naltrexone (NTX) in interpenetrating network of HMW-CS IH and LMW-CS IH.

**Figure 10:**
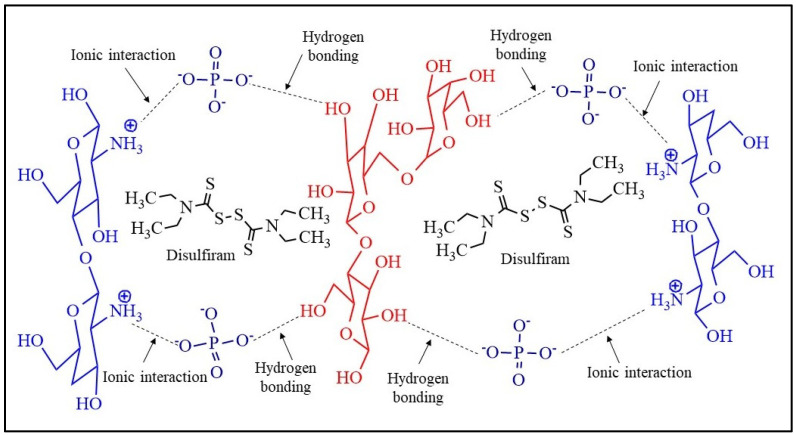
Loading of disulfiram (DSF) in interpenetrating network of HMW-CS IH and LMW-CS IH.

The formation of intermolecular hydrogen bonds and ionic interactions leads to a physically crosslinked hydrogel. The developed IPN of hydrogel incorporated the drugs, NTX and DSF, as illustrated in Figure 9 and Figure 10. 

### 2.9. Drug Release 

**Figure 11:**
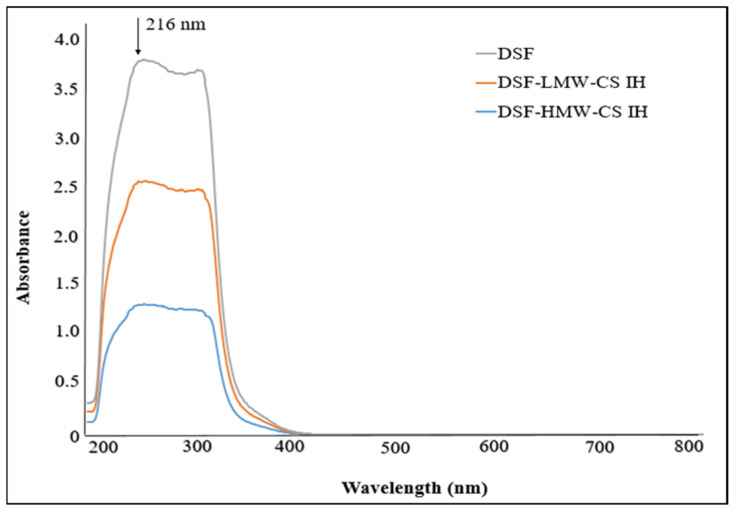
UV spectra of DSF encapsulated in LMW-CS IH and HMW-CS IH.

**Figure 12:**
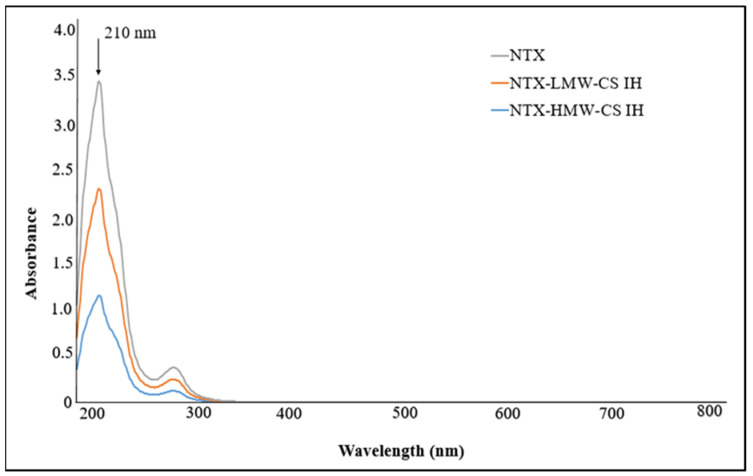
UV spectra of NTX encapsulated in LMW-CS IH and HMW-CS IH.

Each drug’s sustained release impact was determined by measuring absorbance at a certain wavelength using an UV–visible spectrophotometer. Figure 11 corresponds to λmax = 216 for DSF, while Figure 12 shows λmax = 210 for NTX. 

**Figure 13:**
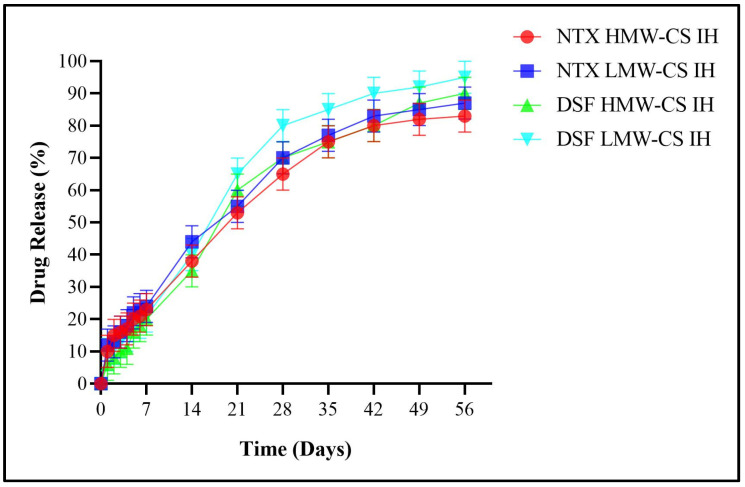
Drug release profile for HMW- and LMW-CS hydrogels in PBS (pH 7.4) at 37 °C temperature.

The gradual diffusion of DSF and NTX from the interconnected 3D structure of the hydrogels was in synchronization with the hydrogen bonding between the amino groups of CS and the hydroxyl groups of GG [47]. The release of DSF and NTX from their corresponding IHs in PBS is shown in Figure 13. 

The crosslinking strength was higher in HMW-CS IH, resulting in NTX release of up to 22% and DSF release up to 30% within 7 days. In comparison, at day 7, the LMW-CS IH released 40% NTX and 35% DSF. After 14 days, the HMW-CS IH released 44% of NTX and 47% of DSF, and the LMW-CS IH released 59% of NTX and 50% of DSF with a weak crosslinked structure. After 28 days, the HMW-CS IH released 62% of NTX and 72% of DSF in PBS, and LMW-CS IH released 70% of NTX and 80% of DSF. Overall, the sustained-release effect of NTX and DSF was achieved [37]. After 56 days, the HMW-CS IH provided a prolonged release of antinarcotic drugs, with NTX released by up to 78% and DSF up to 85%, and the LMW-CS IH released NTX by up to 85% and DSF up to 90%. There were non-significant differences observed among different formulations for drug release in PBS. 

According to the biodegradation analysis, the HMW-CS IH degraded by 55% and the LMW-CS IH degraded by 68% of their original weight at the end of 56 days. The desired therapeutic levels of NTX and DSF were achieved within this time period. The degree of crosslinking was higher in HMW-CS IH, which can be attributed to the increased deacetylation of HMW-CS and the subsequent increase in the formation of ionic and hydrogen bond interactions. The drugs (NTX and DSF) were loaded in the IPN of both HMW-CS IH and LMW-CS IH. When the drug-loaded hydrogels were placed in the release medium, the polymer slowly eroded and the crosslinking network broke down. The drugs were released earlier from LMW-CS IH than HMW-CS IH due to the lower crosslinking of LMW-CS with GG.

For the first 7 days, HMW-CS IH released NTX at 3.14% (0.08 mg) and DSF at 4.3% (0.11 mg) daily, while LMW-CS IH released NTX at 5.72% (0.14 mg) and DSF at 5% (0.13 mg) daily. In the following 7 days, HMW-CS IH released NTX at 3.14% (0.08 mg) and DSF at 2.43% (0.06 mg) daily, whereas LMW-CS IH released NTX at 4.14% (0.10 mg) and DSF at 1.43% (0.04 mg) daily. For the subsequent 14 days, HMW-CS IH released NTX at 1.3% (0.03 mg) and DSF at 0.93% (0.02 mg) daily, while LMW-CS IH released NTX at 0.79% (0.02 mg) and DSF at 2.14% (0.05 mg) daily. After 28 days, HMW-CS IH released NTX at 0.57% (0.01 mg) and DSF at 0.46% (0.01 mg), while LMW-CS IH released NTX at 0.53% (0.01 mg) and DSF at 0.35% (0.009 mg) daily. The levels of the encapsulated antinarcotics remained stable until 56 days, which will help patients addicted to narcotics adhere to the treatment. Initially, the drug release was high, followed by consistent levels being achieved thereafter.

The sustained release of NTX and DSF was observed for up to 56 days due to the biodegradation time of HMW-CS IH and LMW-CS IH observed in the current study. Furthermore, the sustained-release effect was correlated with the biodegradation of the IH. However, the HMW-CS IH underwent low biodegradation due to increased crosslinking caused by the ionic linkages and hydrogen bond formation.

**Figure 14:**
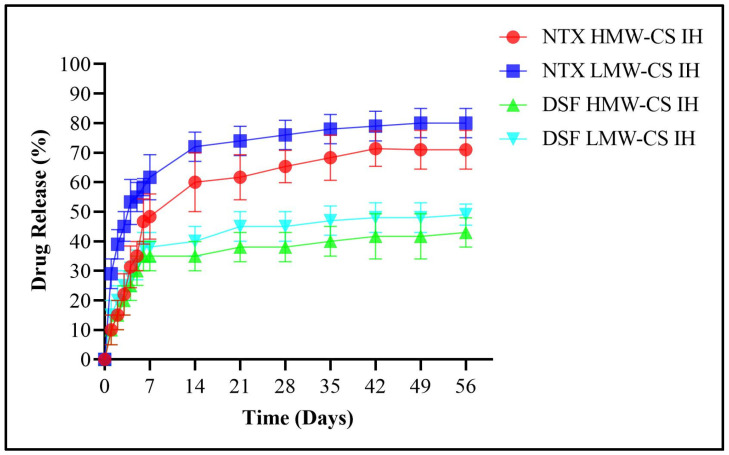
DSF and NTX release in 0.3% ethanol.

**Figure 15:**
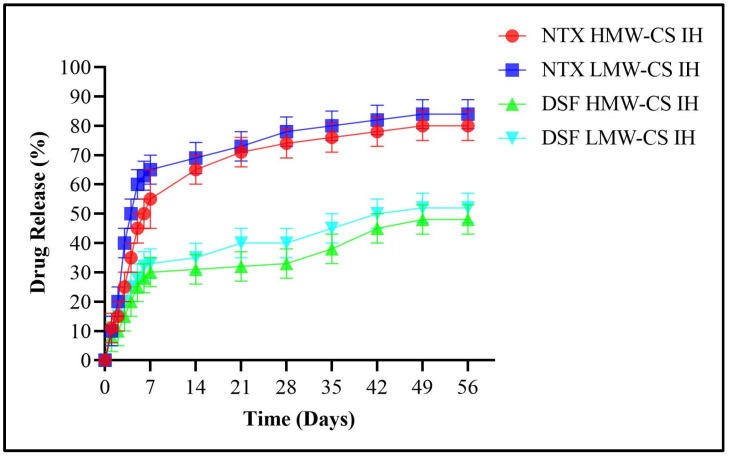
DSF and NTX release in 0.4% ethanol.

**Figure 16:**
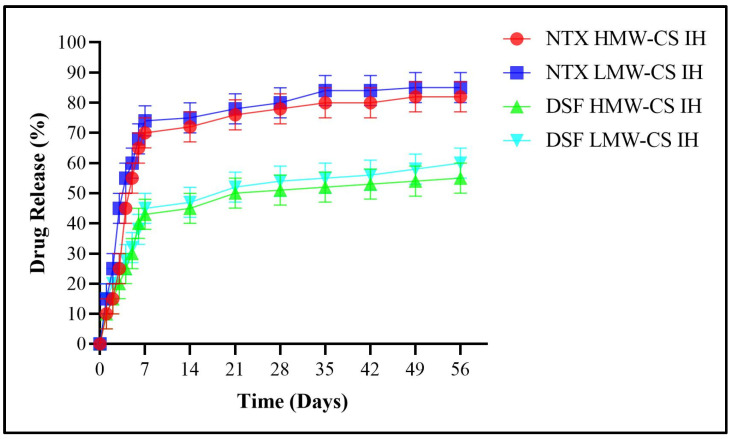
DSF and NTX release in 0.5% ethanol.

An analysis was conducted on the release of NTX and DSF in ethanol solutions with concentrations of 0.3%, 0.4%, and 0.5%, as depicted in Figure 14, Figure 15 and Figure 16, respectively. The HMW-CS IH and LMW-CS IH loaded with NTX and DSF were placed at a temperature of 37 °C and agitation of 100 rpm. The drug release was monitored over a 2-month period. Initially, there was robust release until 7 days. After day 7, the release was smooth but a significant difference was observed between NTX- and DSF-loaded formulations. The HMW-CS IH released NTX at a maximum level of 82% in 0.5% ethanol and a minimum level of 78% in 0.3% ethanol. The LMW-CS IH released NTX at a maximum rate of 85% in 0.5% ethanol and a minimum rate of 80% in 0.3% ethanol. The HMW-CS IH released 55% DSF in 0.5% ethanol and minimum 47% DSF in 0.3% ethanol. The LMW-CS IH released 60% of DSF in 0.5% ethanol and 50% DSF in 0.3% ethanol. 

The degree of CS deacetylation and the charge on drug molecules affect the level of drug release in surrounding medium [48]. The degree of deacetylation was lower in LMW-CS IH, where the NTX and DSF release was observed to be higher. On the contrary, a high degree of deacetylation controlled the release of NTX and DSF in HMW-CS IH. The positive charge on the encapsulated NTX allowed increased interaction with the surrounding ethanol molecules and increased release from the interpenetrating network of the hydrogel, and DSF molecules were uncharged and their interaction with ethanol was to a lower extent. Heredia, N.S. et al.; 2022 developed 3D bio-printed scaffolds and demonstrated drug release on the basis of crosslinking density and swelling capacity. The rate of drug release was influenced by the amount of poly ethylene glycol employed and consequent crosslinking by the functional groups of the crosslinker acrylate [49].

### 2.10. Drug Release Kinetics

The drug release kinetic models for NTX release in PBS from HMW-CS IH and LMW-CS IH are depicted in Supplementary Figure S1. NTX release from HMW-CS IH fitted to the zero-order kinetics and Korsmeyer–Peppas models with an R^2^ (coefficient of determination) value of 0.9508 and 0.9806, respectively, whereas the R^2^ value was found to be slightly lower (0.8631) for the first-order kinetics of NTX release from HMW-CS IH. Similarly, NTX release from LMW-CS IH followed zero-order kinetics with an R^2^ value of 0.9455 and the Korsmeyer–Peppas model with an R^2^ value of 0.9776. For NTX release from LMW-CS IH, the R^2^ value of 0.8532 was noted for the first-order drug kinetics model. 

The drug release kinetics models for DSF release in PBS from HMW-CS IH and LMW-CS IH are depicted in Supplementary Figure S2. DSF release from HMW-CS IH fitted to zero-order kinetics with an R^2^ value of 0.9455 and the Korsmeyer–Peppas model with an R^2^ value of 0.9839. The DSF release from HMW-CS IH revealed a lower R^2^ value of 0.8216 for first-order kinetics. Similarly, DSF release from LMW-CS IH followed zero-order kinetics with an R^2^ value of 0.9293 and the Korsmeyer–Peppas model with an R^2^ value of 0.9718. However, the DSF release from LMW-CS IH yielded a low R^2^ value of 0.842 for first-order kinetics. The R^2^ values of the developed IHs were found to be closer to unity for the zero-order kinetics and Korsemeyer–Peppas models, which confirmed the drug release from the IHs with erosion and diffusion in a sustained manner in the surrounding PBS release medium.

The drug release kinetics models for NTX release in 0.3% ethanol are depicted in Supplementary Figure S3. The R^2^ value of NTX release from HMW-CS IH for the Korsemeyer–Peppas model was 0.8667. The zero-order and first-order kinetics yielded the R^2^ values of 0.7381 and 0.5442, respectively. Similarly, NTX release from LMW-CS IH exhibited an R^2^ value of 0.8396 for the Korsemeyer–Peppas model and lower R^2^ values of 0.596 and 0.496 for zero-order and first-order kinetics, respectively. 

The drug release kinetics models for DSF release in 0.3% ethanol are depicted in Supplementary Figure S4. The DSF release from HMW-CS IH followed the Korsemeyer–Peppas model with an R^2^ value of 0.8341. DSF HMW-CS IH yielded lower R^2^ values of 0.6882 and 0.519 for zero-order and first-order drug release, respectively. In a similar manner, DSF release from LMW-CS IH followed the Korsemeyer–Peppas model with an R^2^ value of 0.8912, and low R^2^ values of 0.6951 and 0.5707 for zero-order and first-order drug release were noticed. 

The drug release kinetics models for NTX release in 0.4% ethanol are shown in Supplementary Figure S5. The NTX release from HMW-CS IH followed the Korsemeyer–Peppas model with an R^2^ value of 0.8199. The zero-order and first-order drug release kinetics models yielded the R^2^ values of 0.6319 and 0.4735, respectively, for NTX release from HMW-CS IH. The R^2^ values for NTX release from LMW-CS IH for the zero-order, first-order, and Korsemeyer–Peppas models were found to be 0.5183, 0.3285, and 0.6679, respectively, in 0.4% ethanol.

The drug release kinetics models for DSF release in 0.4% ethanol are depicted in Supplementary Figure S6. The DSF release from HMW-CS IH yielded R^2^ values for the zero-order, first-order, and Korsemeyer–Peppas models of 0.8263, 0.6249, and 0.8875, respectively. The R^2^ values of DSF release from LMW-CS IH for the zero-order, first-order, and Korsemeyer–Peppas models were found to be 0.8281, 0.6358, and 0.9153, respectively. 

The drug release kinetics models for NTX release in 0.5% ethanol are depicted in Supplementary Figure S7. For NTX release from HMW-CS IH, the zero-order, first-order, and Korsemeyer–Peppas models yielded lower R^2^ values of 0.5441, 0.3955, and 0.7483, respectively. In a similar manner, the NTX release from LMW-CS IH yielded R^2^ values of 0.5407, 0.3844, and 0.7367 for the zero-order, first-order, and Korsemeyer–Peppas models, respectively. 

The drug release kinetics models for DSF release in 0.5% ethanol are depicted in Supplementary Figure S8. For DSF release from HMW-CS IH, lower R^2^ values of 0.6607, 0.5176, and 0.8571 were noticed for the zero-order, first-order, and Korsemeyer–Peppas models, respectively. In a similar manner, the DSF release from LMW-CS IH in 0.5% ethanol fitted to the Korsemeyer–Peppas model with an R^2^ value of 0.9204. For zero-order and first-order kinetics, the R^2^ values of 0.7431 and 0.6159 were noticed. 

For NTX and DSF release in ethanol (0.3%, 0.4%, and 0.5%), the value of R^2^ was found to be consistently higher for the Korsemeyer–Peppas model in comparison to zero-order and first-order drug release kinetics. The model’s acceptance criteria was based on the R^2^ value, with the highest value indicating the best fit for drug release in the Korsmeyer–Peppas model [49]. Initially, drug release occurred through diffusion from weak crosslinking points in the hydrogel, followed by the erosion of the hydrogel matrix [50]. Once these weak crosslinking points were fully opened, the remaining drug release was attributed to the biodegradation of the hydrogel, which was conducted for a period of 56 days. An initial, robust release of the enclosed drug was necessary to achieve the desired therapeutic concentration of NTX and DSF. Subsequently, the drug levels were maintained through the gradual erosion of the polymer matrix, following the Korsemeyer–Peppas model for drug release kinetics [51]. It was confirmed that the drugs were released from the polymeric matrix of the IHs and a combination of release mechanisms were involved for the release of NTX and DSF from the developed IH formulations. 

The parameters for drug release kinetics, as mentioned in Supplementary Figures S1–S8, are shown in Table 1. K_o_ refers to the constant for zero-order kinetics, K is the constant for first-order kinetics, and n is the diffusion exponent for the Korsemeyer–Peppas model. The values of n between 0.45 and 0.89 refer to the non-Fickian drug release from the formulation to the surrounding release medium [52]. Kasiński, A., et al. 2020 conducted a similar study where controlled release of 5-fluorouracil from hydrogel based on poly(ether-ester) was confirmed by fitting to zero-order kinetics and the Korsmeyer–Peppas model [53]. In another study performed by Rasool, A., et al., 2020, the non-Fickian release of lidocaine from carrageenan and alginate-based hydrogel blends was investigated by studying the drug release kinetics models on lidocaine release in PBS [54]. 

The prepared formulations of NTX HMW-CS IH, NTX LMW-CS IH, DSF HMW-CS IH, and DSF LMW-CS IH yielded n values of 0.5773, 0.5765, 0.7554, and 0.6866, respectively, for drug release in PBS. The n values showed all the developed IHs followed a non-Fickian drug release mechanism in PBS. For 0.3%, 0.4%, and 0.5% ethanol, NTX release from HMW-CS IH followed non-Fickian release, with n values of 0.4665, 0.4587, and 0.4589, respectively. However, NTX release from LMW-CS IH and DSF release from HMW-CS IH and LMW-CS IH yielded n values of less than 0.45, and thus followed Fickian drug release, in 0.3%, 0.4%, and 0.5% ethanol solutions [55]. 

## 3. Materials and Methods

### 3.1. Materials

Chemicals used were HMW-CS (MW ≈ 900 kDa, Purity ≈ 98.5%, degree of deacetylation ≥90%), LMW-CS (MW ≈ 100.45 kDa, purity ≈98.5%, degree of deacetylation ≥ 80%), GG (spray dried), sodium bi phosphate dibasic (98%), NTX (98%, MW 341.4), DSF (97%, MW 297), nicotine (99%), and ethanol. All chemicals were acquired from Sigma-Aldrich and were of analytical grade.

### 3.2. Preparation of Hydrogel

Both LMW- and HMW-CS (125 mg/5 mL) were dissolved separately in 0.1 N HCl at room temperature. The dissolution process involved stirring at 100–150 rpm for 60 min [56]. The GG was dissolved in deionized water at room temperature using a quantity of 2 g/5 mL and stirred at 100–150 rpm for 60 min. The LMW- and HMW-CS solutions were separately added dropwise into the GG solution at room temperature and stirred at 100 rpm for 10 min. Then, 0.5 mL of sodium bi phosphate dibasic solution was added dropwise at room temperature and stirred at 100 rpm for a further 10–15 min [57]. The composition of the prepared hydrogels is shown below in Table 2.

### 3.3. Fourier Transform Infrared (FT-IR) Spectroscopy

The FT-IR analysis of LMW-CS IH and HMW-CS IH was performed using an A2 Technologies’ portable attenuated reflectance (ATR) Fourier Transform Infrared Spectroscopy (ATRS-FTIR) spectrometer. Samples were vacuum-dried and ground prior to analysis. The materials were analyzed using a spectrometer with a resolution of 4 cm, covering the range of 500 cm^−1^ to 4000 cm^−1^. The FT-IR spectrum of each CS hydrogel was acquired by analyzing 1 mg of dried material on the sensor of the instrument and comparing its spectra to the spectra of CS and GG [58]. 

### 3.4. Thermo-Gravimetric Analysis (TGA) 

The HMW-CS IH and LMW-CS IH were examined under different temperature settings using TGA analysis. The IHs (4–5 mg) were weighed in aluminum pans and the pans were sealed with a perforated lid. Measurements were collected from 0 to 800 °C at a heating rate of 10 °C per minute. All measurements were performed under nitrogen gas (purity 99.99%) with a flow rate of 70 mL per minute [59].

### 3.5. Scanning Electron Microscopy (SEM)

The prepared LMW-CS IH and HMW-CS IH were dried in a hot-air oven overnight at a temperature of 40 °C. The electronic beam was stammered onto the dried sample, and images were taken at resolutions of 10 µm, 5 µm, 1 µm, and 500 nm [60].

### 3.6. Rheology

Rheological tests were performed on 1 g of LMW-CS IH and HMW-CS IH using a MCR 301 Rheometer. Oscillatory rheology tests were conducted to determine the strength of the hydrogel. An oscillatory frequency sweep test was conducted within the frequency range of 0.1–100 Hz with a constant strain of 1%. This study focused on analyzing the elements of the complex modulus, including the storage modulus (G′) and the loss modulus (G″) [61]. 

### 3.7. Swelling Analysis

The water absorption capacity of the HMW-CS IH and LMW-CS IH was assessed using swelling tests conducted at a pH of 1.2 (gastric), 7 (deionized water), 7.4 (physiological), and 8.5 (intestinal). The dry hydrogel (0.1 g) was placed in 30 mL of the respective solution in vials at room temperature. The hydrogel samples were taken out from the solutions at regular intervals of 30 min, dried to eliminate excess water using tissue paper, and then weighed on an electronic balance [62,63]. These experiments were performed in triplicate. The swelling percentage was calculated by Equation (1), given below.

Swelling percentage = (Ws − Wd)/Wd × 100
(1)

‘Ws’ = weight of the swollen hydrogel and ‘Wd’ = weight of the dry hydrogel. 

### 3.8. Biodegradation Analysis

For the analysis of biodegradation, 0.1 g of HMW-CS IH and LMW-CS IH were placed separately in PBS solution (pH 7.4, temperature 37 °C, and 100 rpm). The hydrogel was taken out of the PBS solution at specific time period (1–14 days daily, then after 14 days an interval of 7 days till 56 days) and weighed on an electronic balance [64]. This experiment was conducted in triplicate. The percentage of weight loss was calculated by using Equation (2), as given below.

Weight loss percentage = (Wi − Wt)/Wi × 100
(2)

‘Wi’ = initial weight and ‘Wt’ = remaining weight of hydrogel.

### 3.9. Drug Loading

NTX (2.5 mg) was dissolved in 250 µL 0.1 N HCl and DSF (2.5 mg) was dissolved in 250 µL of ethanol. The NTX and DSF were separately incorporated into HMW-CS IH and LMW-CS IH at room temperature and 100 rpm before the addition of the crosslinker [65].

### 3.10. Drug Release 

Drug release was checked in PBS and ethanol with different concentrations (0.3%, 0.4%, and 0.5%). The HMW-CS IH and LMW-CS IH, loaded with the medicinal substances (NTX and DSF), were immersed in 30 mL PBS and the ethanol solutions in 50 mL tubes. They were then subjected to shaking at a rate of 100 rpm and a temperature of 37 °C. The solution (1 mL) was periodically (1–7 days daily, then after 7 days at intervals of 7 days till 56 days) withdrawn for drug release analysis and substituted with fresh PBS solution to keep the volume constant, and the absorbance (λmax) of the encapsulated drugs (NTX and DSF) was measured using an UV spectrophotometer [66].

### 3.11. Drug Release Kinetics

The drug release data points in the PBS and ethanol solutions (0.3%, 0.4%, and 0.5%) were then subjected to zero-order, first-order, and Korsmeyer–Peppas mathematical models according to the equations mentioned below.

Zero-order

Mt = M_o_ + K_o_ t (3)

First-order

logC = logC_o_ − Kt/2.303 (4)

Korsmeyer–Peppas model

lnMt/M_o_ = n lnt + lnK (5)

Mt is the amount of drug released at time t, M_o_ is initial drug concentration, and K_o_ and K are the rate constants of zero-order and first-order drug release. For the Korsmeyer–Peppas model, n is the diffusion exponent, which correlates to the drug release mechanism [54]. 

### 3.12. Statistical Analysis

All the data were collected in triplicate and are presented as mean ± standard error. Statistical analysis of the data was performed by analysis of variance (ANOVA) using Graph Pad Prism 9.5.1 software. A *p* value < 0.05 was considered to be statistically significant among means of the compared samples. 

## 4. Conclusions

Hydrogels with injectable qualities were produced by mixing GG with low and high molecular weight CS and linking them using sodium phosphate dibasic. The HMW-CS IH, due to the availability of a higher number of protonated amino groups, underwent increased crosslinking with GG. The thermal stability, mechanical strength, and rheological properties of HMW-CS IH were higher than those of LMW-CS IH. Both HMW-CS IH and LMW-CS IH successfully encapsulated the antinarcotics DSF and NTX and provided sustained release over 56 days in PBS and ethanol. The release of NTX and DSF in PBS was fitted to zero-order kinetics and Korsemeyer–Peppas models that confirmed the release of NTX and DSF in a sustained manner from the IHs composed of biopolymers (CS and GG) via a non-Fickian release mechanism. The positive charge on the surface of NTX enhanced its release in ethanol solutions at concentrations of 0.3%, 0.4%, and 0.5%. To understand the better efficacy of these IHs, large-scale in vivo studies should be conducted to evaluate their pharmacokinetic and therapeutic efficacy. These in vivo studies could provide valuable information and insight related to drug stability, metabolism, and biocompatibility with body tissues. 

## Figures and Tables

**Table 1:** NTX and DSF release analysis from HMW-CS IH and LMW-CS IH.

Formulation	Release Medium	Zero-Order Kinetics		First-Order Kinetics		Korsemeyer–Peppas Model		Drug Release Mechanism
		R^2^	K_o_	R^2^	K	R^2^	n	
NTX HMW-CS IH	PBS	0.9508	0.0147	0.8631	0.0159	0.9806	0.5773	Non-Fickian
NTX LMW-CS IH	PBS	0.9455	0.0152	0.8532	0.0158	0.9776	0.5765	Non-Fickian
DSF HMW-CS IH	PBS	0.9455	0.0168	0.8216	0.0203	0.9839	0.7554	Non-Fickian
DSF LMW-CS IH	PBS	0.9293	0.0178	0.842	0.0188	0.9178	0.6866	Non-Fickian
NTX HMW-CS IH	Ethanol (0.3%)	0.7381	0.0105	0.5442	0.0109	0.8667	0.4665	Non-Fickian
NTX LMW-CS IH	Ethanol (0.3%)	0.5965	0.0067	0.496	0.005	0.8396	0.2202	Fickian
DSF HMW-CS IH	Ethanol (0.3%)	0.6882	0.0049	0.519	0.0074	0.8341	0.3197	Fickian
DSF LMW-CS IH	Ethanol (0.3%)	0.6951	0.0049	0.5707	0.0063	0.8912	0.2688	Fickian
NTX HMW-CS IH	Ethanol (0.4%)	0.6319	0.0104	0.4735	0.0103	0.8199	0.4587	Non-Fickian
NTX LMW-CS IH	Ethanol (0.4%)	0.5183	0.0085	0.3285	0.0077	0.6679	0.3755	Fickian
DSF HMW-CS IH	Ethanol (0.4%)	0.8263	0.0062	0.6249	0.01	0.8875	0.4043	Fickian
DSF LMW-CS IH	Ethanol (0.4%)	0.8281	0.0064	0.6358	0.0088	0.9153	0.3606	Fickian
NTX HMW-CS IH	Ethanol (0.5%)	0.5441	0.0099	0.3955	0.0098	0.7483	0.4589	Non-Fickian
NTX LMW-CS IH	Ethanol (0.5%)	0.5407	0.0087	0.3844	0.0073	0.7367	0.3437	Fickian
DSF HMW-CS IH	Ethanol (0.5%)	0.6607	0.0067	0.5176	0.0089	0.8571	0.3884	Fickian
DSF LMW-CS IH	Ethanol (0.5%)	0.7431	0.0069	0.6159	0.0079	0.9204	0.3269	Fickian

**Table 2:** Composition of HMW-CS IH and LMW-CS IH.

Formulation	Chitosan (CS)	Guar Gum (GG)	Crosslinker	Crosslinking
HMW-CS IH	125 mg HMW-CS/5 mL 0.1 N HCl	2 g/5 mL deionized water	Sodium bi phosphate dibasic	Physical crosslinking
LMW-CS IH	125 mg LMW-CS/5 mL 0.1 N HCl	2 g/5 mL deionized water	Sodium bi phosphate dibasic	Physical crosslinking

## Data Availability

Data is contained within article.

## Supplementary Materials

The following supporting information can be downloaded at: https://www.mdpi.com/article/10.3390/ph17060749/s1, Figure S1. The drug release kinetics models in PBS; (a) zero order kinetics, (b) first order kinetics, and (c) Korsmeyer-Peppas model for NTX loaded HMW-CS IH, and (d) zero order kinetics, (e) first order kinetics, and (f) Korsmeyer-Peppas model for NTX loaded LMW-CS IH. Figure S2. The drug release kinetics models in PBS; (a) zero order kinetics, (b) first order kinetics, and (c) Korsmeyer-Peppas model for DSF loaded HMW-CS IH, and (d) zero order kinetics, (e) first order kinetics, and (f) Korsmeyer-Peppas model for DSF loaded LMW-CS IH. Figure S3. The drug release kinetics models in 0.3% ethanol; (a) zero order kinetics, (b) first order kinetics, and (c) Korsmeyer-Peppas model for NTX loaded HMW-CS IH, and (d) zero order kinetics, (e) first order kinetics, and (f) Korsmeyer-Peppas model for NTX loaded LMW-CS IH. Figure S4. The drug release kinetics models in 0.3% ethanol; (a) zero order kinetics, (b) first order kinetics, and (c) Korsmeyer-Peppas model for DSF loaded HMW-CS IH, and (d) zero order kinetics, (e) first order kinetics, and (f) Korsmeyer-Peppas model for DSF loaded LMW-CS IH. Figure S5. The drug release kinetics models in 0.4% ethanol; (a) zero order kinetics, (b) first order kinetics, and (c) Korsmeyer-Peppas model for NTX loaded HMW-CS IH, and (d) zero order kinetics, (e) first order kinetics, and (f) Korsmeyer-Peppas model for NTX loaded LMW-CS IH. Figure S6. The drug release kinetics models in 0.4% ethanol; (a) zero-order kinetics, (b) first order kinetics, and (c) Korsmeyer-Peppas model for DSF loaded HMW-CS IH, and (d) zero-order kinetics, (e) first order kinetics, and (f) Korsmeyer-Peppas model for DSF loaded LMW-CS IH. Figure S7. The drug release kinetics models in 0.5% ethanol; (a) zero order kinetics, (b) first order kinetics, and (c) Korsmeyer-Peppas model for NTX loaded HMW-CS IH, and (d) zero order kinetics, (e) first order kinetics, and (f) Korsmeyer-Peppas model for NTX loaded LMW-CS IH. Figure S8. The drug release kinetics models in 0.5% ethanol; (a) zero order kinetics, (b) first order kinetics, and (c) Korsmeyer-Peppas model for DSF loaded HMW-CS IH, and (d) zero order kinetics, (e) first order kinetics, and (f) Korsmeyer-Peppas model for DSF loaded LMW-CS IH.
