# Supplementary material for: Design and Characterization of Chitosan-Based Smart Injectable Hydrogel for Improved Sustained Release of Antinarcotics
Source: Pharmaceuticals (Basel). 2024 Jun 7;17(6):749. doi: 10.3390/ph17060749 (PMC11206616; doi:10.3390/ph17060749)
Supplement: Supplementary file 1 [file pharmaceuticals-17-00749-s001.zip › pharmaceuticals-3011513-supplementary.pdf]

Supplementary Data for Drug Release Kinetics

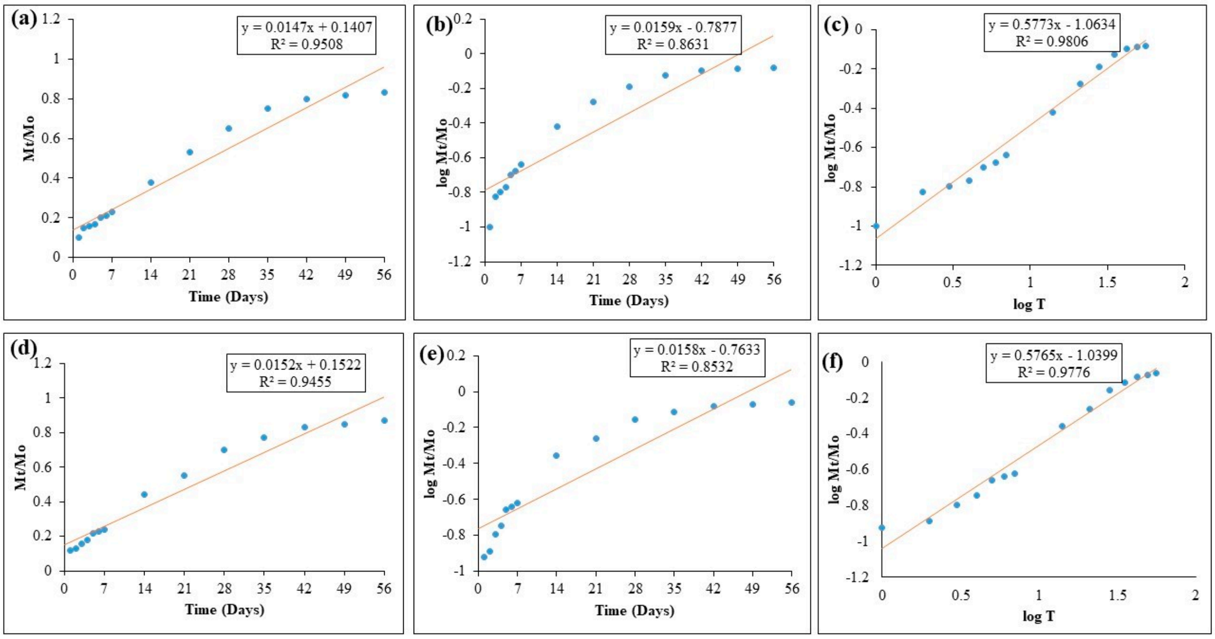

Figure S1. The drug release kinetics models in PBS; (a) zero order kinetics, (b) first order kinetics, and (c) Korsmeyer-Peppas model for NTX loaded HMW-CS IH, and (d) zero order kinetics, (e) first order kinetics, and (f) Korsmeyer-Peppas model for NTX loaded LMW-CS IH.

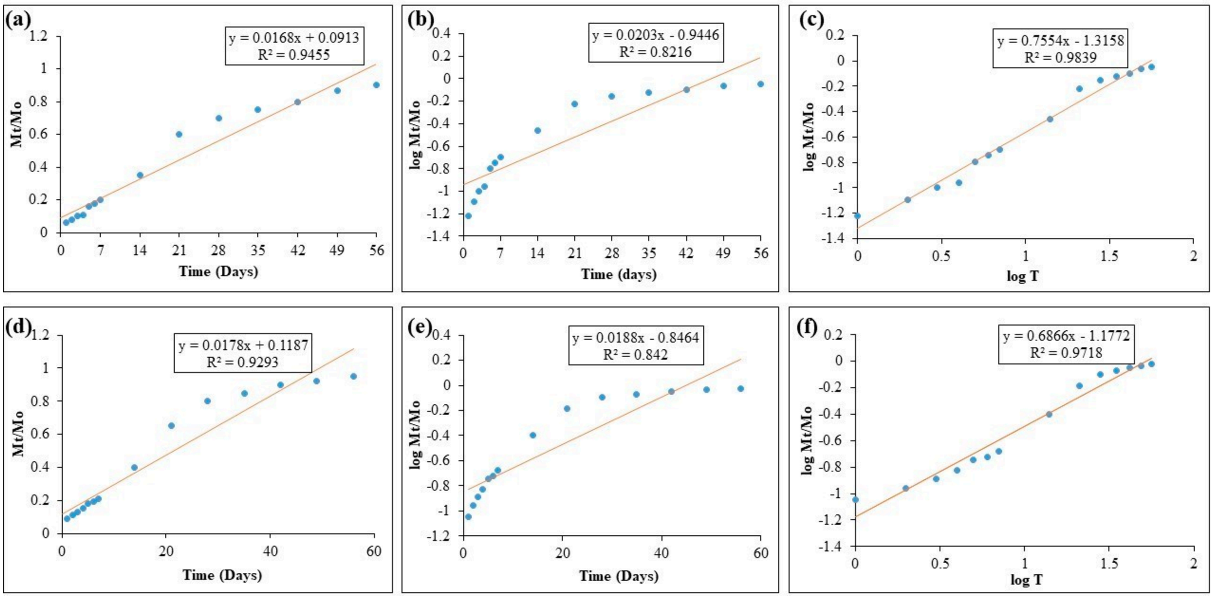

Figure S2. The drug release kinetics models in PBS; (a) zero order kinetics, (b) first order kinetics, and (c) Korsmeyer-Peppas model for DSF loaded HMW-CS IH, and (d) zero order kinetics, (e) first order kinetics, and (f) Korsmeyer-Peppas model for DSF loaded LMW-CS IH.

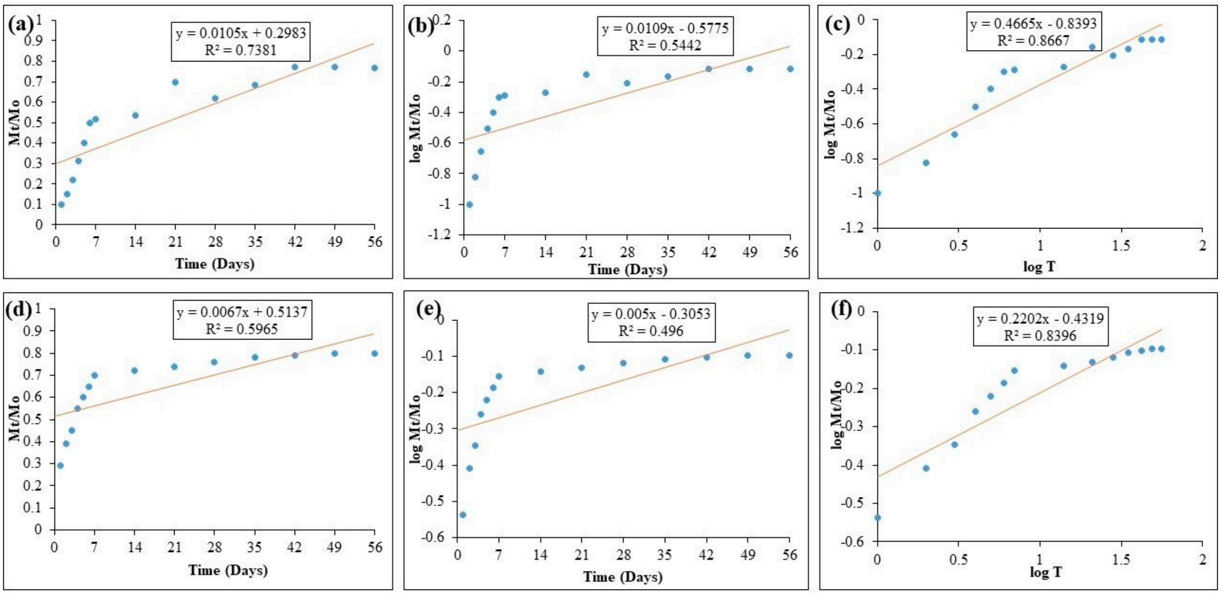

Figure S3. The drug release kinetics models in 0.3% ethanol; (a) zero order kinetics, (b) first order kinetics, and (c) Korsmeyer-Peppas model for NTX loaded HMW-CS IH, and (d) zero order kinetics, (e) first order kinetics, and (f) Korsmeyer-Peppas model for NTX loaded LMW-CS IH

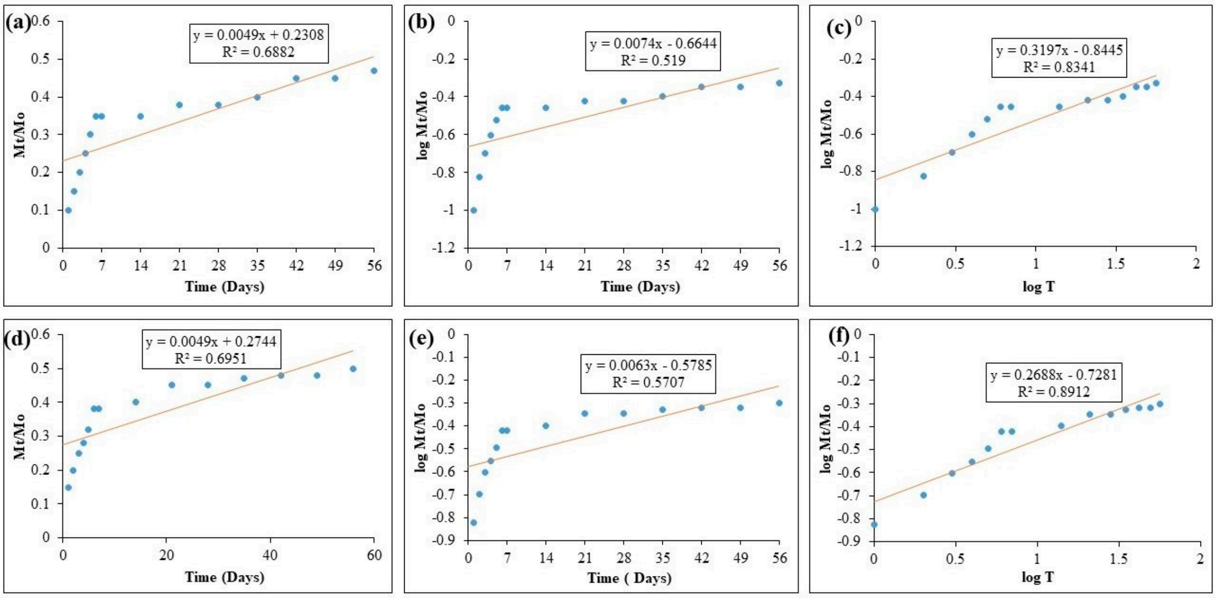

Figure S4. The drug release kinetics models in 0.3% ethanol; (a) zero order kinetics, (b) first order kinetics, and (c) Korsmeyer-Peppas model for DSF loaded HMW-CS IH, and (d) zero order kinetics, (e) first order kinetics, and (f) Korsmeyer-Peppas model for DSF loaded LMW-CS IH.

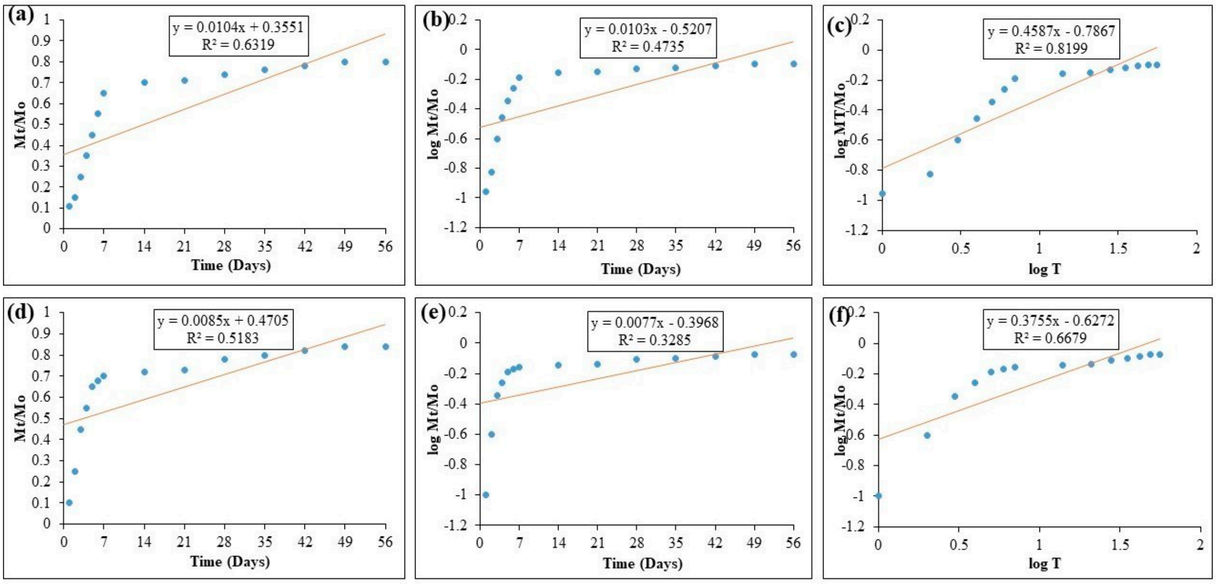

Figure S5. The drug release kinetics models in 0.4% ethanol; (a) zero order kinetics, (b) first order kinetics, and (c) Korsmeyer-Peppas model for NTX loaded HMW-CS IH, and (d) zero order kinetics, (e) first order kinetics, and (f) Korsmeyer-Peppas model for NTX loaded LMW-CS IH.

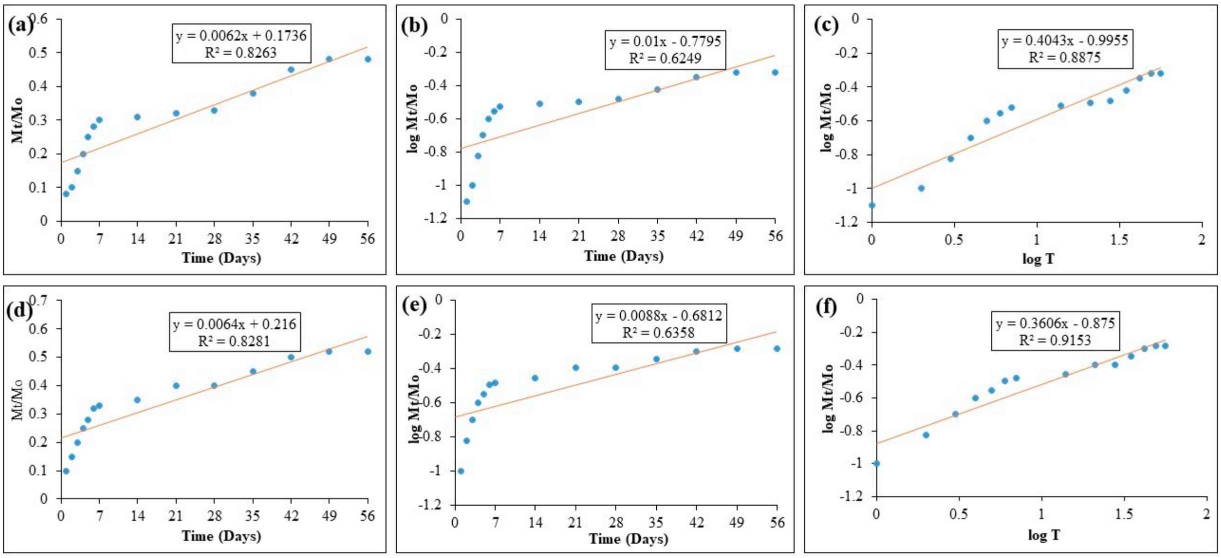

Figure S6. The drug release kinetics models in 0.4% ethanol; (a) zero-order kinetics, (b) first order kinetics, and (c) Korsmeyer-Peppas model for DSF loaded HMW-CS IH, and (d) zero-order kinetics, (e) first order kinetics, and (f) Korsmeyer-Peppas model for DSF loaded LMW-CS IH.

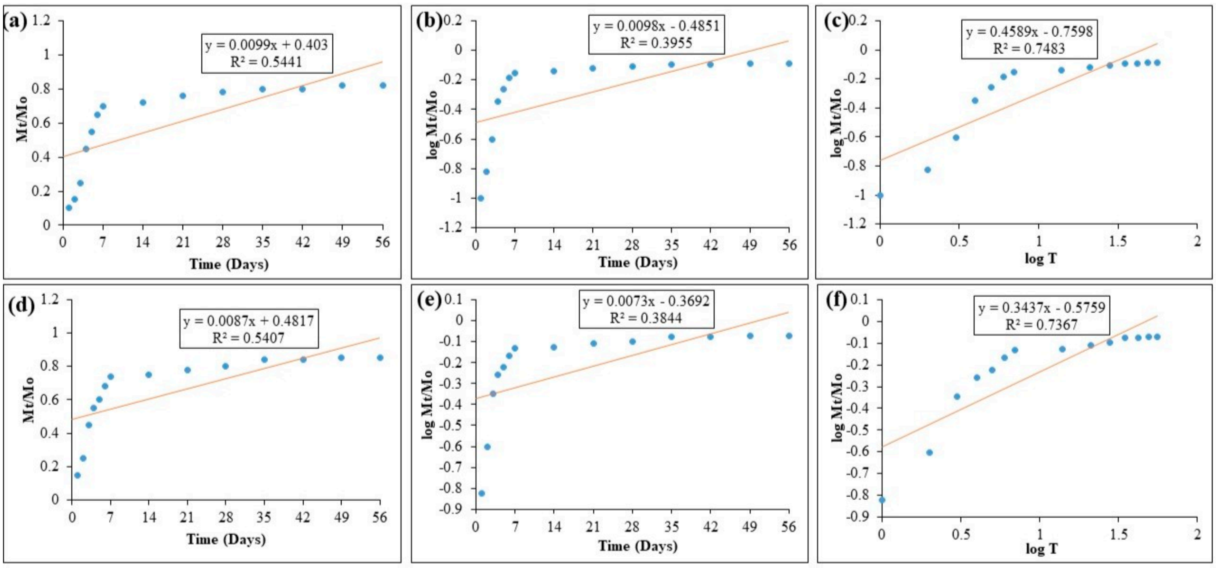

Figure S7. The drug release kinetics models in 0.5% ethanol; (a) zero order kinetics, (b) first order kinetics, and (c) Korsmeyer-Peppas model for NTX loaded HMW-CS IH, and (d) zero order kinetics, (e) first order kinetics, and (f) Korsmeyer-Peppas model for NTX loaded LMW-CS IH.

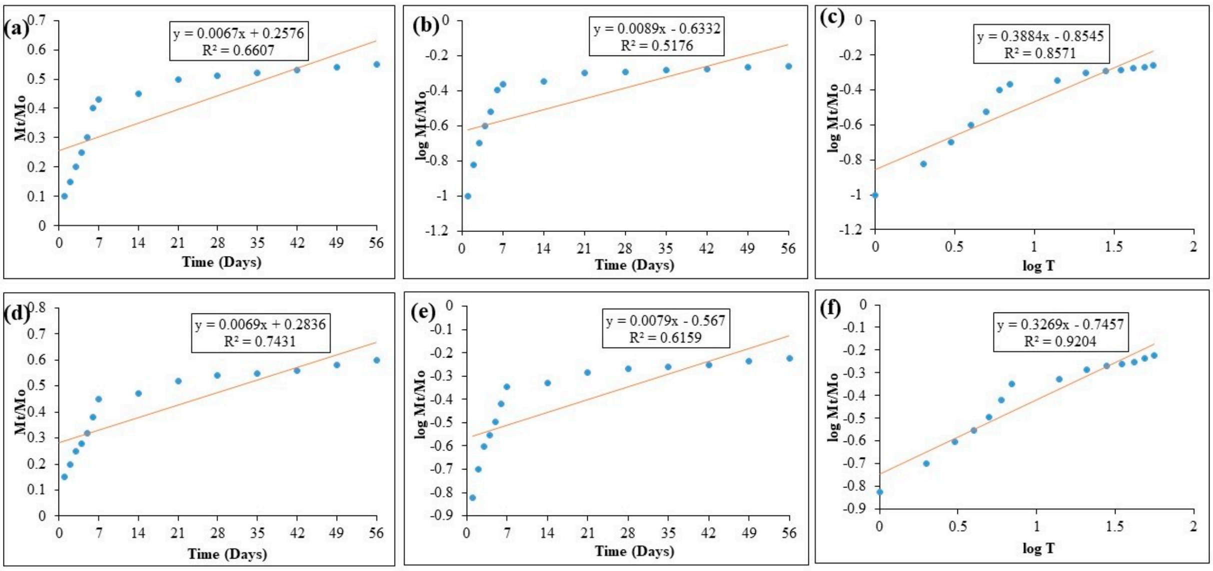

Figure S8. The drug release kinetics models in 0.5% ethanol; (a) zero order kinetics, (b) first order kinetics, and (c) Korsmeyer-Peppas model for DSF loaded HMW-CS IH, and (d) zero order kinetics, (e) first order kinetics, and (f) Korsmeyer-Peppas model for DSF loaded LMW-CS IH.
